# Technology Delivered Interventions for Depression and Anxiety in Children and Adolescents: A Systematic Review and Meta-analysis

**DOI:** 10.1007/s10567-018-0271-8

**Published:** 2018-09-18

**Authors:** Rebecca Grist, Abigail Croker, Megan Denne, Paul Stallard

**Affiliations:** 1 Department for Health, University of Bath, 6.19 Wessex House, Bath, BA2 7AY UK; 2 Oxford Health NHS Foundation Trust, Child, and Adolescent Mental Health Service, Temple House, Keynsham, UK; 3 School of Applied Social Science, University of Brighton, Mayfield House, Falmer, Brighton, BN1 9PH UK

**Keywords:** Depression, Anxiety, Child, Adolescent, Technology, Review

## Abstract

Depression and anxiety are common during adolescence. Whilst effective interventions are available treatment services are limited resulting in many adolescents being unable to access effective help. Delivering mental health interventions via technology, such as computers or the internet, offers one potential way to increase access to psychological treatment. The aim of this systematic review and meta-analysis was to update previous work and investigate the current evidence for the effect of technology delivered interventions for children and adolescents (aged up to 18 years) with depression and anxiety. A systematic search of eight electronic databases identified 34 randomized controlled trials involving 3113 children and young people aged 6–18. The trials evaluated computerized and internet cognitive behavior therapy programs (CBT: *n* = 17), computer-delivered attention bias modification programs (ABM: *n* = 8) cognitive bias modification programs (CBM: *n* = 3) and other interventions (*n* = 6). Our results demonstrated a small effect in favor of technology delivered interventions compared to a waiting list control group: *g* = 0.45 [95% CI 0.29, 0.60] *p* < 0.001. CBT interventions yielded a medium effect size (*n* = 17, *g* = 0.66 [95% CI 0.42–0.90] *p* < 0.001). ABM interventions yielded a small effect size (*n* = 8, *g* = 0.41 [95%CI 0.08–0.73] *p* < 0.01). CBM and ‘other’ interventions failed to demonstrate a significant benefit over control groups. Type of control condition, problem severity, therapeutic support, parental support, and continuation of other ongoing treatment significantly influenced effect sizes. Our findings suggest there is a benefit in using CBT based technology delivered interventions where access to traditional psychotherapies is limited or delayed.

## Introduction

Anxiety and depression in children and young people are common (Merikangas et al. 2009). Over a six-month period up to 8% of adolescents suffer from a major depressive disorder and cumulatively, by the age of 18, up to 20% will experience at least one clinically significant depressive episode (Costello et al. 2006; Merry et al. 2012a). Recurrence is common with up to 75% experiencing a subsequent episode of depression within 5 years (Lewinsohn et al. 2000). Adolescent depression has a negative impact on relationships, developmental trajectories, schooling, and educational attainment, and increases the risk of attempted and completed suicide (Birmaher et al. 1996; Fletcher 2008; Gould et al. 2003). Similarly, up to 10% of children and 20% of adolescents will suffer from an anxiety disorder (Essau et al. 2012). Anxiety disorders are associated with poor academic performance and adversely affect relationships, along with increasing the risk of depression, illicit drug dependence and educational under-achievement in young adulthood (Kim-Cohen et al. 2003; Woodward and Fergusson 2001).

Psychological therapies are effective in the treatment of anxiety and depressive disorders in children and adolescents (James et al. 2013; Reynolds et al. 2012). However, many children and young people with mental health problems do not receive specialist treatments (Merikangas et al. 2011; Ford et al. 2005). Despite initiatives to increase the capacity of child mental health services to deliver evidence-based interventions demand for face to face therapy continues to outstrip capacity (Fonagy et al. 2017). This has led to interest in the use of information and communications technology (e-mental health) to increase capacity to support and improve the mental health of children and young people (Riper et al. 2010; Boydell et al. 2014; Ebert et al. 2015; Hollis et al. 2017).

E-mental health embraces a range of digital technologies that deliver interventions via computers, or through web-based platforms via mobile tablets or smartphones (Hollis et al. 2017). Advantages of digital technologies include greater reach to geographically isolated populations; flexible access; increased convenience; fewer visits to specialist clinics; greater privacy and anonymity; enhanced treatment fidelity; rapid scalability; and low-cost delivery (Clarke et al. 2015; MacDonell and Prinz 2017). There are also several concerns about the use of digital technologies including the absence of a strong motivating and supportive therapeutic relationship; negative professional attitudes to their use; technology failure; questions about their effectiveness in treating severe mental health problems; high rates of attrition; and concerns about data security and quality control (Lal and Adair 2014).

It has been suggested that digital technology may be particularly appealing to adolescents who are typically early adopters and regular users of new technologies (Johnson et al. 2015). In the UK, 83% of 12–15-year olds have their own smartphone, 55% have their own tablet, with 99% going online for almost 21 h per week (Ofcom 2013). In the US, 93% of 12–17-year olds have access to a desktop or laptop computer with 74% having internet access (Madden et al. 2013). Whilst the development of technology to support mental health interventions with children is still in its infancy, results from studies with adults have shown that internet and computer-delivered interventions can be effective for the treatment of depression and anxiety (Andersson and Cujipers 2009; Andrews et al. 2010; Griffiths et al. 2010).

Although e-mental health interventions are fast-developing, those specifically developed for children with anxiety and depression are more limited (Richardson et al. 2010; Calear and Christensen 2010; Pennant et al. 2015). Small RCTs have demonstrated that established evidence-based face to face CBT anxiety interventions such as Cool Teens can be effective when delivered via a CD-ROM with minimal therapist support (Wuthrich et al. 2012). Similarly, online CBT anxiety programs such as BRAVE were found to be very acceptable to young people and as effective as face to face CBT (Spence et al. 2011). In terms of depression, encouraging results have been reported for Stressbusters, a computerized CBT program (Smith et al. 2015; Wright et al. 2017) and a computer game (SPARX) used both as an intervention and as a prevention program (Merry et al. 2012a, b; Perry et al. 2017).

Novel interventions including attentional bias modification training (ABM) and cognitive bias modification (CBM) which attempt to reduce the attentional and cognitive biases associated with depression and anxiety have also been investigated (Bar-Haim et al. 2011; LeMoult et al. 2017). In a review, Pennant et al. (2015) note that although ABM and CBM improved the outcomes of attention and interpretation bias, no conclusive benefits were found for depression and anxiety outcomes. Further investigation into these interventions is therefore warranted.

Systematic reviews have explored the efficacy of digital technologies in the treatment of depression and anxiety disorders in children and adolescents. The first reviews published in 2010 identified only a handful of very small studies resulting in the authors being unable to draw any conclusions other than noting that this appears to be a promising area to explore (Calear and Christensen 2010; Richardson et al. 2010). More recent reviews identified more studies but included young adults (up to the age of 25) and included both prevention and treatment studies (Hollis et al. 2017; Pennant et al. 2015). Although the authors were cautiously positive they noted that the evidence was inconclusive, and that the research suffered from several methodological limitations. The review by Pennant et al. (2015) was updated by Hollis et al. (2017) and included studies up until 1/11/2015. However, given the small number of anxiety and depression treatment studies identified during the update period, the authors did not undertake any specific analysis of these problem groups. Given that E-health is a rapidly developing area we do not have any contemporary systematic analysis of the effects of technological interventions for children and young people (up to age 18) with depression or anxiety.

This review will address this issue and will conduct a meta-analysis exploring the effect of technological interventions for children and young people up to the age of 18 with anxiety and depression. We will undertake sub-group analyses to investigate the effects of anxiety and depression, control condition, problem severity, theoretical basis, therapist assistance, and parental involvement, and whether other interventions were also provided.

## Method

### Study Identification

Eight electronic databases were systematically searched for publications between January 2013 and September 2017. These included: APA PsychNET, Embase, Google Scholar, PubMed, Science Direct, Scopus, Social Policy and Practice and Web of Science. Databases of main journals JMIR, Cyberpsychology, Behavior and Social Networking and Internet Interventions were also searched using key search terms. A systematic search of each database was conducted using a combination of search terms relating to the mental health problems targeted, the medium of intervention delivery (computerized, internet, smartphone), population age (child, teenage, adolescent), and the type of study. Database-specific filters such as human population, English language, and age groups were applied where available. Appendix A contains the full list of search strings by databases. Reference lists of included articles were also screened for potentially relevant studies. One researcher (R.G.) conducted the systematic identification, screening, and checking for eligibility of full-text articles for inclusion. This process was then independently conducted by two researchers (A.C/ and M.D.) with disagreements discussed and a consensus on inclusion or exclusion reached.

### Inclusion Criteria

We included randomized controlled trials of technology delivered psychological interventions for use by children and adolescents for depression or anxiety disorders. Studies were included if the sample was 18 years old or under. Studies with over 18’s were included if the mean age of the sample was 18 or less. We included studies if the sample were assessed to have a diagnosed anxiety or depression disorder or had elevated symptoms which were of mild to moderate severity. This must have been diagnosed by a clinician or assessed by the research team by diagnostic interview or screening for cut off scores on an anxiety or depression questionnaire. The intervention needed to be primarily delivered via technology such as computers, CD-ROM, the internet, smartphones, or virtual reality. Technologies which only augmented traditional face to face therapies or did not constitute a significant proportion of the delivery were excluded. Only randomized controlled trials (RCT) were included. As per previous work (e.g., Pennant et al. 2015), there were no restrictions placed on the theoretical basis of the intervention. We included RCT’s which compared an intervention to gold standard face to face CBT, other therapeutically ‘active’ conditions, attention/placebo training conditions and wait-list controls.

### Exclusion Criteria

Studies were excluded if they did not meet the inclusion criteria described. Studies of universal preventative interventions and studies in which the mental health of the sample was not screened were excluded. Articles were also excluded if the intervention was primarily aimed at parents of children with depression or anxiety and did not involve a component aimed at the children. Interventions in which therapists provided ‘live’ therapy over the internet either via video conferencing or instant messaging were excluded.

### Data Extraction

Study information including study characteristics, participant information, and mental health outcomes was extracted and included in an Excel spreadsheet. Study characteristics included authors, year of publication, the country in which the study was conducted, and sample sizes. Study characteristics also included how participants were recruited and whether participants were permitted to continue ongoing pharmacological or psychological interventions. Participant information included sample age, primary mental health problem and whether this was screened as elevated or fully diagnosed. To be consistent with the age classification adopted by Pennant et al. (2015) which reflects the transition in the UK from primary to secondary school, studies were classified as focusing solely on children (aged 5–11 years), adolescents (12–18 years), or a mixed age group. Program details included the program name, theoretical basis, where the program was delivered and whether there was any parent support.

Information on therapist support was also extracted. This involved classifying each program according to therapist support as outlined by Newman et al. (2011). Programs could either be: (1) self-administered (SA—therapist contact for assessment at most); (2) predominantly self—administered (PSH—giving initial therapeutic rationale, direction on how to use the program and periodic check-ins, < 90 min of time); and(3) minimal contact therapy (MCT—active involvement of therapist, help in applying specific therapeutic techniques, > 90 min of time). Studies were categorized according to therapist support only. Therefore, interventions that provided technical assistance, but not therapeutic support were categorized as self-administered.

For the meta-analysis, the mental health outcomes were the means (*M*) and standard deviations (*SD*) on the primary outcome measure of anxiety and/or depression symptoms at post-intervention. When trials used a wait-list control condition (WLC) and an ‘active’ control condition (such as individual or group CBT) outcomes from both conditions were extracted. Outcomes from the WLC were used as the comparator in the main meta-analysis and sub-group analyses. A specific sub-group analysis was undertaken to compare technological delivered interventions against different categories of control condition. Information on program completion was extracted by obtaining the number of participants who were allocated to and completed the intervention condition within the allotted time frame.

### Quality Assessment

The quality of each study was assessed according to the Cochrane Collaboration’s Risk of Bias Tool. This was conducted by one researcher and then checked by two researchers independently. Disagreements were discussed and resolved to meet a consensus. Studies were assigned either ‘low risk’, ‘unclear risk’, or ‘high risk’ status regarding several domains. These included: selection bias, performance bias, detection bias, attrition bias, reporting bias and final ‘other’ category of identifiable biases.

### Data Analysis

The statistical software package Review Manager Version 5.3 (Review Manager; The Cochrane Collaboration 2014) was used to conduct the meta-analysis. Post-intervention means standard deviations and sample sizes were entered for the intervention and control conditions of each study. A random-effects meta-analysis was conducted using the standardized mean differences (SMD) to calculate effect size (Hedges’ *g*). To calculate the heterogeneity of effect sizes the Q statistic and *I*^2^ statistic was used. A significant Q statistic implies significant heterogeneity indicating more variation in effect sizes that can be attributed to chance alone. The *I*^2^ statistic expresses the heterogeneity as a percentage, with values of 25% associated with low heterogeneity, 50% moderate and 75% high heterogeneity (Crombie and Davies 2009).

Sub-group analyses were conducted to investigate the influence of (1) control condition (face to face CBT, other therapeutically active control, attention/placebo control and wait-list), (2) mental health problem (anxiety or depression), (3) problem severity (confirmed diagnosis or elevated symptoms), (4) theoretical basis of intervention (CBT, ABM, CBM, other), (5) therapist support (SH, PSH, MCT), (6) active parental involvement (yes or no) and (7) continuation of other treatment for depression or anxiety (yes or no).

## Results

### Study Selection

**Fig. 1:**
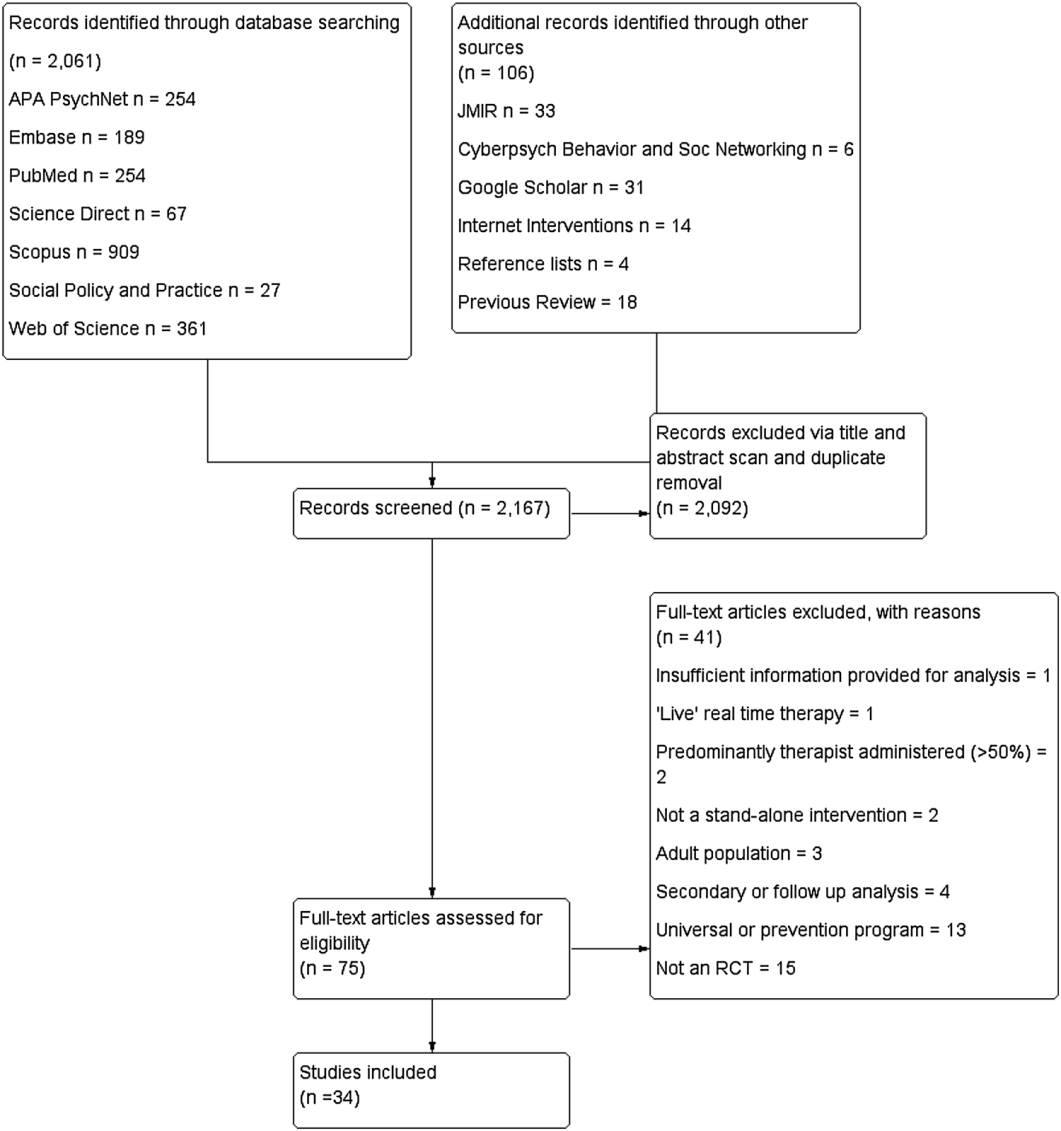
PRISMA flow diagram of results and publication selection

The systematic literature search yielded 2167 results, of which 2092 were excluded based on screening the abstract, title and duplicate removal. A total of 75 full-text articles were assessed for inclusion; 41 were excluded leaving 34 studies for inclusion in the meta-analysis. Figure 1 details the results at each stage and reasons for exclusion.

### Study Characteristics

Tables 1 and 2 contain the main study characteristics and references of studies included in the meta-analysis contains full references of all included studies. Altogether, the 34 studies included 3113 children and adolescents (*n* = 1517 in intervention conditions and *n* = 1596 in control conditions) with sample sizes ranging between 19 and 257. Participants were aged between 6 and 18 years old. Some studies (*n* = 5) included participants over 18 (maximum age 22) however for each study the mean age of the sample was under 18. For the other studies, three involved a study sample of children (5–12 years), 18 involved an adolescent population (13–18 years) with the remaining 13 studies involving a mix of children and adolescents (5–18 years). Studies were conducted in the Netherlands (*n* = 8), Australia (*n* = 8), China (*n* = 3), Sweden (*n* = 3), the UK (*n* = 3), the USA (*n* = 2), Israel (*n* = 2), New Zealand (*n* = 2), Canada (*n* = 1), Ireland (*n* = 1), and Thailand (*n* = 1).

**Table 1:** Selected study characteristics

Study	Country	Age of sample (years)	Sample size	Diagnostic status	Referral	Intervention setting	Primary outcome	Continuing other treatment
Bar-Haim et al. (2011)	Israel	10	35	Elevated (Anx)	Subsample of larger RCT	University site	STAIC	NR
Conaughton et al. (2017)	Australia	8–12	42	Diagnosed (Anx)	Professional and self-referral	Home/AWI	CSR on the ADIS for DSMIV	Psychological—No Pharmacological—NR
De Voogd et al. (2017)	Amsterdam (Netherlands)	11–19	70	Elevated (Anx and Dep)	School	Home	SCARED (Anx) CDI (Dep)	NR
Fitzgerald et al. (2016)	Ireland	15–18	120	Elevated (Anx)	School	School	SPAI-C	No
Fu et al. (2013)	China	12–17	28	Diagnosed (Anx)	Professional	MH centre	Negative mood on VASs derived from PANAS-C	NR
Hoek et al. (2012)	Amsterdam (Netherlands)	12–21 (m = 16.1 (2.3)	45	Elevated (Dep and Anx)	Professional and self-referral	Home/AWI	CES-D (Dep) HADS-A (Anx)	Psychological—No Pharmacological—NR
Ip et al. (2016)	China	13–17	257	Elevated (Dep)	School	Home/AWI	CESD-R	Psychological—NR Pharmacological—No
Le Moult et al. (2017)	USA	7–13	46	Diagnosed (Dep)	Self - referred	Home and university site	CDI	NR
Lenhard et al. (2017)	Sweden	12–17	67	Diagnosed (OCD)	Professional and self-referral	Home/AWI	CY-BOCS	Yes (pharmacological)
March et al. (2009)	Australia	7–12	73	Diagnosed (Anx)	Professional and self-referral	Home/AWI	CSR on the ADIS for DSMIV	No
Merry et al. (2012a, b)	New Zealand	12–19 (m = 16.6, SD = 1.6)	187	Elevated (Dep)	Professional	Primary care health sites	CDRS-R	No
Muris et al. (1998)	Netherlands	8–17	26	Diagnosed (Spider Phobia-Anx)	Self-referred	University site	SPQ-C	NR
Pergamin-Hight et al. (2016)	Israel	6–18	67	Diagnosed (SAD)	Self-referred	University site	CSR on the ADIS for DSMIV	No
Poppelaars et al. (2016)	Netherlands	11–16	101	Elevated (Dep)	School	Home/AWI	RADS-2	No
Rickhi et al. (2015)	Canada	13–18	31	Diagnosed (Dep)	Professional and self-referral	Home/AWI	CDRS-R	Yes
Schleider and Weisz (2017)	USA	12–15	96	Elevated (Dep and Anx)	Self-referral	University site	CDI (Dep) SCARED-C (Anx)	NR
Scholten et al. (2016)	Netherlands	11–15	138	Elevated (Anx)	School	School	SCAS	No
Schoneveld et al. (2016)	Netherlands	8–13	124	Elevated (Anx)	School	School	SCAS	No
Schoneveld et al. (2017)	Netherlands	7–12	174	Elevated (Anx)	School	School	SCAS	No
Smith et al. (2015)	UK	12–16	112	Elevated (Dep)	School	School	MFQ	Yes
Spence et al. (2011)	Australia	12–18	115	Diagnosed (Anx)	Professional and self-referral	Home/AWI	CSR on the ADIS for DSMIV	NR
Spence et al. (2017)	Australia	8–17	125	Diagnosed (SAD)	Professional and self-referral	Home/AWI	CSR on the ADIS for DSMIV	No
Sportel et al. (2013)	Netherlands	14–16	240	Elevated (SAD)	School	Home/AWI	RCADS social phobia	NR
Stallard et al. (2011)	UK	11–17	20	Diagnosed (Anx and Dep)	Professional	Home and School	The AWS (Dep) SCAS-C (Anx)	NR
Stasiak et al. (2014)	New Zealand	13–18	34	Elevated (Dep)	School counsellor	School	CDRS-R	No
Tillfors et al. (2011)	Sweden	15–21 years (M = 16.5, SD = 1.6)	19	Diagnosed (SAD)	Self-referred and school	Home/AWI	SPSQ-C	Yes (pharmacological)
Vigerland et al. (2016a)	Sweden	8–12	93	Diagnosed (Anx)	Self-referred	Home/AWI	CSR on the ADIS for DSMIV	Yes (pharmacological)
Wannachaiyakul et al. (2017)	Thailand	14–22	84	Elevated (Dep)	Professional	Youth detention centre	The Thai version Patient Health Questionnaire (PHQ-9)	No
Waters et al. (2013)	Australia	7–13	37	Diagnosed (Anx)	NR	Home	CSR on the ADIS for DSMIV	NR
Waters et al. (2015)	Australia	6–12	59	Diagnosed (Anx)	Self-referred	Home	CSR on the ADIS for DSMIV	No
Waters et al. (2016)	Australia	6–12	41	Diagnosed (Anx)	Self-referred	Home	CSR on the ADIS for DSMIV	No
Wright et al. (2017)	UK	12–18	91	Elevated (Dep)	Professional	Community, OP or school	MFQ	NR
Wuthrich et al. (2012)	Australia	14–17	43	Diagnosed (Anx)	Professional and self-referral	Home	CSR on the ADIS for DSMIV	Yes (pharmacological)
Yang et al. (2016)	China	12–18	45	Diagnosed (Dep)	School	NR	HAM-D	NR

Diagnostic status: *Anx* anxiety, *Dep* depression, *OCD* obsessive compulsive disorder, *SAD* social anxiety disorder. Intervention setting: *AWI* anywhere with internet, *OP* outpatient, *MH* mental health, *NR* not reported. Primary outcome: CSR on the ADIS for *DSMIV* Clinician Severity Rating (CSR) on the Anxiety Disorders Interview Schedule for DSMIV, *SCARED* Screen for Child Anxiety Related Emotional Disorders, *SPAI-C* Screen for Child Anxiety Related Emotional Disorders, *CESD-R* Centre for Epidemiologic Studies Depression Scale, *CY-BOCS* Children’s Yale-Brown Obsessive Compulsive Scale, *RADS-2* Reynolds Adolescent Depression Scale, *CDRS-R* Children’s Depression Rating Scale-Revised, *SCAS* Spence Children’s Anxiety Scale, *SCAS-C* Spence Children’s Anxiety Scale Child Version, *MFQ* Mood and Feelings Questionnaire, *HAM-D* Hamilton Depression Scale, *HADS-A* Hospital Anxiety and Depression Scale-Anxiety Subscale, *SCARED-C* Screen for Child Anxiety and Related Disorders-Child Version

**Table 2:** Summary of extracted program and study information

Study	Intervention	Target problem	Therapist support	Parental support	Intervention (*n* =)	Intervention description	Control (*n* =)	Control description	Program completion
ICBT/CCBT									
Conaughton et al. (2017)	BRAVE-ONLINE	Anxiety	MCT	Yes	21	Weekly 60 min sessions, 10 child and six parent sessions + 2 booster sessions	21	WLC	19%
Ip et al. (2016)	Grasping the opportunity (CATCH-IT)	Depression	SA	No	130	Ten sessions designed to improve negative cognition Reduce negative behaviors and strengthen resiliency	127	Anti-smoking website (APC)	20%
Lenhard et al. (2017)	BiP OCD	OCD	MCT	Yes	33	12 Chapters that contain texts to read, films and animations. ERP is the main treatment component	34	WLC	27%
March et al. (2009)	BRAVE-ONLINE	Anxiety	MCT	Yes	30	10 Weekly 60 min sessions Parents 6 weekly 60 min sessions	29	WLC	88%
Merry et al. (2012a, b)	SPARX	Depression	SA	No	94	Interactive fantasy game 7 weekly modules	93	TAU (OTC)	87%
Muris et al. (1998)	Computerized spider exposure	Anxiety—spider phobia	SA	No	8	Hierarchically structured computerized spider exposure. 2.5 h single session	9	EMDR (OTC)	NR
Poppelaars et al. (2016)	SPARX	Depression	SA	No	51	Fantasy game with seven levels, CBT principles are introduced through challenges, interactions with a guide and homework tasks	50 and 51	School based CBT and WLC	75%
Smith et al. (2015)	Stressbusters	Depression	SA	No	55	Eight modules, 30–45 min. Interactive presentation featuring videos, animations, graphics and printouts	57	WLC	86%
Spence et al. (2011)	BRAVE-ONLINE	Anxiety	MCT	Yes	44	See previous description for BRAVE-ONLINE	44 and 27	F2F CBT and WLC	39%
Spence et al. (2017)	No name (SAD-CBT)	SAD	MCT	Yes	47	10 Weekly 60 min sessions + 2 booster sessions. PE, PSS, RT GE	30	WLC	NR
Stallard et al. (2011)	Think feel do	Anx and Dep	MCT	NR	6	Six 30–45 min sessions over 6 weeks, CD-ROM based CBT workbook	9	WLC	60%
Stasiak et al. (2014)	The journey	Depression	SA	No	17	CD-ROM fantasy game. Seven modules of 30 min	17	Computerized psychoeducation (APC)	94%
Tillfors et al. (2011)	No name	Social anxiety disorder	MCT	No	10	CBT self-help manual, which consisted of nine modules adapted for use via the internet	9	WLC	0%
Vigerland et al. (2016a)	No name	Anxiety	MCT	Yes	46	11 Modules accessed over 10 weeks. Exposure is main focus + PE, CS, PSS	47	WLC	NR
Wannachaiyakul et al. (2017)	No name	Depression	PSH	No	42	6 Weekly 45–60 min sessions. PE, PSS identifying, evaluating, and modifying NATs	42	Usual recreational activities (APC)	100%
Wright et al. (2017)	Stressbusters	Depression	SA	No	45	Eight modules, 30–45 min. Interactive presentation featuring videos, animations, graphics and printouts	46	Websites (APC)	62%
Wuthrich et al. (2012)	Cool teens	Anxiety	MCT	Yes	24	CBT based CD-ROM based on cool kids anxiety management program. 8 × 30 min sessions for 12 weeks	19	WLC	NR
ABMT									
Bar-Haim et al. (2011)	No name	Anxiety	SA	No	18	ABM Dot probe task with face stimuli (angry and neutral): 4 × 1 h sessions over 4 days within a 2 week period	16	Placebo training (APC)	NR
De Voogd et al. (2017)	No name	Anx and Dep	SA	No	38	Eight sessions of online visual search ABMT. 4 blocks of 36 trials. Participants had to find and select the only smiling face in a 4 × 4 grid of negative emotional faces (angry, fearful, and sad)	32 and 38	Placebo training (APC) and WLC	NR
Fitzgerald et al. (2016)	No name	SAD	SA	No	61	4 weekly training sessions using a dot-probe task designed to reduce attention bias to threatening stimuli	59	Placebo training (APC)	92%
Pergamin-Hight et al. (2016)	No name	Social anxiety	SA	No	31	Dot probe task with angry and neutral faces. 160 trials (120 angry-neutral, 40 neutral–neutral). Eight sessions, twice a week for 4 weeks	36	Attention training control (APC)	92%
Waters et al. (2013)	No name	Anxiety	SA	Unclear	18	ABM Dot probe task with face stimuli 12 treatment sessions over 3 weeks	16	Attention training control (APC)	67%
Waters et al. (2015)	No Name	Anxiety	SA	Yes	31	As above, 12 treatment sessions totalling 224 trials	28	WLC	87%
Waters et al. (2016)	No name	Anxiety	SA	Yes	22	As above, 12 treatment sessions totalling 224 trials	19	WLC	NR
Yang et al. (2016)	No name	Depression	SA	No	23	Neutral ABM over 2 weeks for eight sessions (320 trials each) then positive ABM for 2 weeks with four sessions(480 trials each)	22	Placebo training (APC)	91%
Other									
Fu et al. (2013)	CBM-I training	Anxiety	SA	No	16	CBM-I Word fragment completion Single session	12	Neutral training (APC)	NR
Hoek et al. (2012)	Internet problem solving	Anx and Dep	PSH	No	22	I-Problem solving therapy one lesson per week, 5 weeks	23	WLC	29%
Le Moult et al. (2017)	Computerized CBM-I training	Depression	SA	No	24	Ambiguous scenarios that ended with a word fragment that disambiguated the scenario in a positive or neutral direction. 8 randomized blocks of 13 scenarios	22	Neutral training (APC)	NR
Rickhi et al. (2015)	The LEAP project	Depression	SA	No	18	8 week online intervention. Aims to treat depression by using ‘spiritually informed principles’ such as forgiveness, gratitude, compassion	13	WLC	NR
Schleider and Wesiz (2017)	Growth mindset intervention	Anx and Dep	SA	No	48	Single session, 30 min computerized growth mindset session	48	Single session computerized supportive therapy (OTC)	100%
Scholten et al. (2016)	Dojo	Anxiety	SA	No	70	Videogame played six times over three weeks, with two one hour sessions per week. Emotion regulation training and heart rate variability (HRV) biofeedback	68	Control game (APC)	Unclear
Schoneveld et al. (2016)	Mindlight	Anxiety	SA	No	62	Videogame played for 5, 1 h sessions. neurofeedback (EEG) training, exposure training and ABM	62	Control game (APC)	Unclear
Schoneveld et al. (2017)	Mindlight	Anxiety	SA	No	86	Videogame played for 6, 1 h sessions. neurofeedback (EEG) training, exposure training and ABM	88	Coping Cat CBT	88%
Sportel et al. (2013)	No name (CBM-I training)	SAD	SA	No	86	ABM dot probe tasks and CBM-I Word fragment completion. Two sessions 2× a week for 10 weeks	84 and 70	Group CBT and WLC	53%

Intervention: *CATCH-IT* Competent Adulthood Transition with Cognitive Behavioral Humanistic and Interpersonal Training, *SPARX* Smart, Positive, Active, Realistic, X-factor thoughts, *SAD-CBT* social anxiety disorder specific cognitive behavioural therapy. Therapist support: *SA* self -administered, *PSH* predominantly self-help and *MCT* minimal contact therapy. Target problem: *Anx* Anxiety, *Dep* Depression, *OCD* obsessive compulsive disorder, *SAD* social anxiety disorder. Intervention description: *ERP* exposure and response prevention, *PE* psychoeducation, *PSS* problem solving strategies, *RT* relaxation training, *GE* graded exposure, *CS* coping strategies, *NATS* negative automatic thoughts, *ABMT* attention bias modification training, *EEG* electroencephalogram. Control description: *APC* attention/placebo control, *OTC* other therapeutic control, *WLC* wait list control, *TAU* treatment as usual. Program completion: *NR* not reported

In terms of comparison groups (see Table 2) four studies compared the intervention to a face to face CBT group (school-based group CBT, individual CBT and group-based CBT; Poppelaars et al. 2016; Schoneveld et al. 2017; Spence et al. 2011; Sportel et al. 2013). All but the Schoneveld et al. (2017) study also included a wait-list control group. Three studies utilized control groups classified as a ‘other therapeutic control’. These conditions included non-CBT therapeutic content or processes e.g., a single session computerized supportive therapy (Schleider and Wesiz 2017), EMDR (Muris et al. 1998) and treatment as usual, the majority of which was counseling (Merry et al. 2012a, b). Fourteen studies included an attention or placebo comparison group comprised of placebo or neutral attention training (*n* = 8; De Voogd et al. 2017 also included a wait-list control), an anti- smoking website (*n* = 1), computerized psychoeducation program (*n* = 1), video games (*n* = 2), usual recreational activities (*n* = 1), and self-help websites with no CBT content (*n* = 1). Finally, 17 studies compared technology delivered interventions to wait-list control conditions.

Continuation of psychological or pharmacological treatment for depression or anxiety was permitted in seven trials (*n* = 6). Several (*n* = 16) trials explicitly forbade either ongoing psychological or pharmacological treatment or both, during the study. For the remainder of the studies ongoing psychological and pharmacological treatment was not reported (*n* = 12).

### Mental Health Problem Characteristics

The majority of interventions (*n* = 20) targeted anxiety disorders (social anxiety disorder-specific *n* = 5, OCD specific *n* = 1, spider phobia *n* = 1, range of anxiety disorders *n* = 13). Ten interventions targeted depression with four being transdiagnostic interventions targeting both anxiety and depression. Over half of the interventions (*n* = 18) involved participants with a confirmed diagnosis of depression or anxiety with the remainder (*n* = 16) including participants with elevated symptoms of depression or anxiety.

### Intervention Characteristics

Interventions were computerized and internet-based CBT (*n* = 17), attention bias modification training (*n* = 8), cognitive bias modification training (*n* = 3), and other interventions (*n* = 6). The other interventions included an internet-based acceptance and commitment therapy program, problem-solving therapy, video games utilizing neuro-feedback, bio-feedback, and emotion regulation training. Less than half of the programs involved guidance and contact from a therapist (*n* = 9 MCT and *n* = 2 PSH) with most programs being self-administered with no therapist support (*n* = 23). Some programs incorporated some form of parent support (*n* = 9), but the majority did not require any active parental involvement (*n* = 23). For the remainder of the studies, this information was unclear (*n* = 2).

### Computerized and Internet-Based CBT

Several (*n* = 17) studies investigated the use of 13 computerized and internet-based CBT programs for depression and anxiety with five focusing on participants with elevated symptoms of depression: Grasping the Opportunity (CATCH-IT; Ip et al. 2016); SPARX (Merry et al. 2012a, b; Poppelaars et al. 2016); Stressbusters (Smith et al. 2015; Wright et al. 2017), The Journey (Stasiak et al. 2014) and one unnamed guided CCBT program (Wannachaiyakul et al. 2017). Participants with diagnosed anxiety disorders were involved in (*n* = 8) programs: BRAVE-ONLINE (Conaughton et al. 2017; March et al. 2009; Spence et al. 2011); BiP-OCD (Lenhard et al. 2017); Cool Teens (Wuthrich et al. 2012); Think Feel Do (Stallard et al. 2011) and four unnamed programs, two for social anxiety disorder (Spence et al. 2017; Tillfors et al. 2011), one for spider phobia (Muris et al. 1998) and one for children with a range of anxiety disorders (Vigerland et al. 2016a) The majority of programs were therapist-assisted (*n* = 10) with seven including parents.

### Attention Bias Modification Training (ABMT)

The use of attention bias modification training was evaluated in eight trials. One included individuals with a diagnosis of depression, four included individuals diagnosed with anxiety, and three with elevated symptoms of anxiety. Length of ABM training varied from four sessions (Bar-Haim et al. 2011; Fitzgerald et al. 2016), eight sessions (De Voogd et al. 2017; Pergamin-Hight et al. 2016) to 12 sessions (Waters et al. 2013, 2015, 2016; Yang et al. 2016). All the ABMT programs were unguided and did not provide any ongoing clinical support from a therapist. The Waters et al. 2015 and 2016 trials were the only ABMT studies to involve active parental input and were the only ABMT trials to use wait-list controls as a comparison group. All other trials compared ABMT to a placebo training.

### Other Technology-Based Interventions

The remaining nine studies evaluated eight separate programs. Of these, three investigated computer and internet-delivered cognitive bias modification interventions, one for depression (LeMoult et al. 2017), one for anxiety disorders (Fu et al. 2013), and one for social anxiety (Sportel et al. 2013). None of the interventions were therapist or parent assisted.

A trial investigated a ‘spiritually informed’ 8-week internet intervention called The LEAP project (Rickhi et al. 2015). The LEAP program aimed to treat depression by using spiritually informed principles such as forgiveness, gratitude, and compassion. It did not involve any therapist or parent support and was compared to a wait-list control. Adolescents in this trial had a diagnosis of depression and were self-referred. They were permitted to continue ongoing psychological or pharmacological interventions for depression.

One trial investigated a single session 30-min, computer-guided growth mindset intervention (Schleider and Weisz 2017) and another an internet-based (guided) problem-solving therapy intervention (Hoek et al. 2012).

Finally, three trials evaluated two video games used to treat anxiety (Dojo, Scholten et al. 2016 and Mindlight; Schoneveld et al. 2016, 2017). Dojo is a 3D immersive video game specifically designed for reducing anxiety in adolescents and incorporates emotion regulation training and heart rate variability (HRV) biofeedback. Dutch schoolchildren screened to have elevated anxiety symptoms played Dojo or a control game (Rayman) six times over three weeks in a computer room at school. The Mindlight video game incorporates neurofeedback (EEG) training, exposure training, and attention bias modification training, played for 5–6 1-h sessions. Both trials involved school children with elevated anxiety symptoms. In one trial Mindlight was compared to a control game and in the other, to school-based CBT training based on the Coping—Cat program. None of the trials involved therapist guidance or parental support.

### Study Quality

**Fig. 2:**
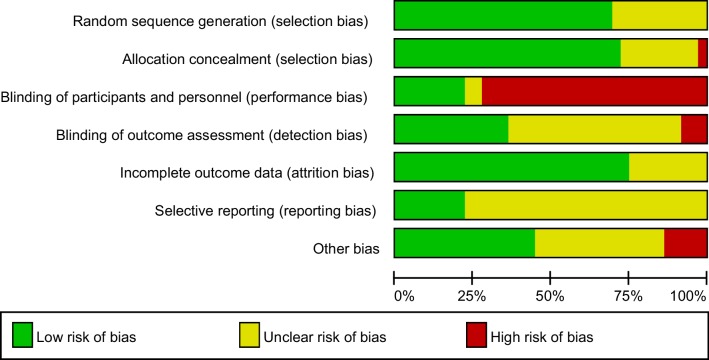
Risk of bias graph. Review authors’ judgements about each risk of bias item presented as percentages across all included studies

Study quality varied as demonstrated in Fig. 2. Most trials (24/34, 71%) adequately reported a random component in sequence generation, mostly using a computer-based random number generator. The remainder did not provide sufficient information to assess selection bias (10/34, 29%). Most trials reported appropriate allocation concealment (25/34, 74%) meaning overall, the risk of selection bias was low. There appeared to be a high risk of performance bias as most of the studies could not ensure blinding of participants and personnel (24/34, 71%). Some studies told participants their group allocation in the first session while other studies were unable to ensure blinding due to the design of the study. Only 13/34 (38%) studies reported adequate blinding of outcome assessment with the majority not providing enough information to assess (unclear risk, 18/34, 53%). Overall, the risk of detection bias was therefore high. Risk of attrition bias tended to be low with the majority of studies using appropriate techniques to handle missing data (25/34, 74%). Only eight studies reported on a study protocol and were assigned low risk of reporting bias (8/34, 24%) with the remainder being assigned an unclear risk of reporting bias (26/34, 76%). Finally, under half of the studies were assigned the low risk of ‘other bias’ (15/34, 44%).

### Program Completion

Studies varied substantially in whether (and how) program completion rates were reported. Program completion was defined as completing all the modules/sessions in the intervention within the allotted study timeframe. For 22 studies (65%) these data were clearly reported in the manuscript text or was extractable from CONSORT flow diagrams. For 12 studies (35%) program completion was not reported or calculable from the provided information. Where reported, program completion rates ranged from 0 to 100% with 64% being the average program completion rate for intervention conditions.

### Meta-analysis

**Fig. 3:**
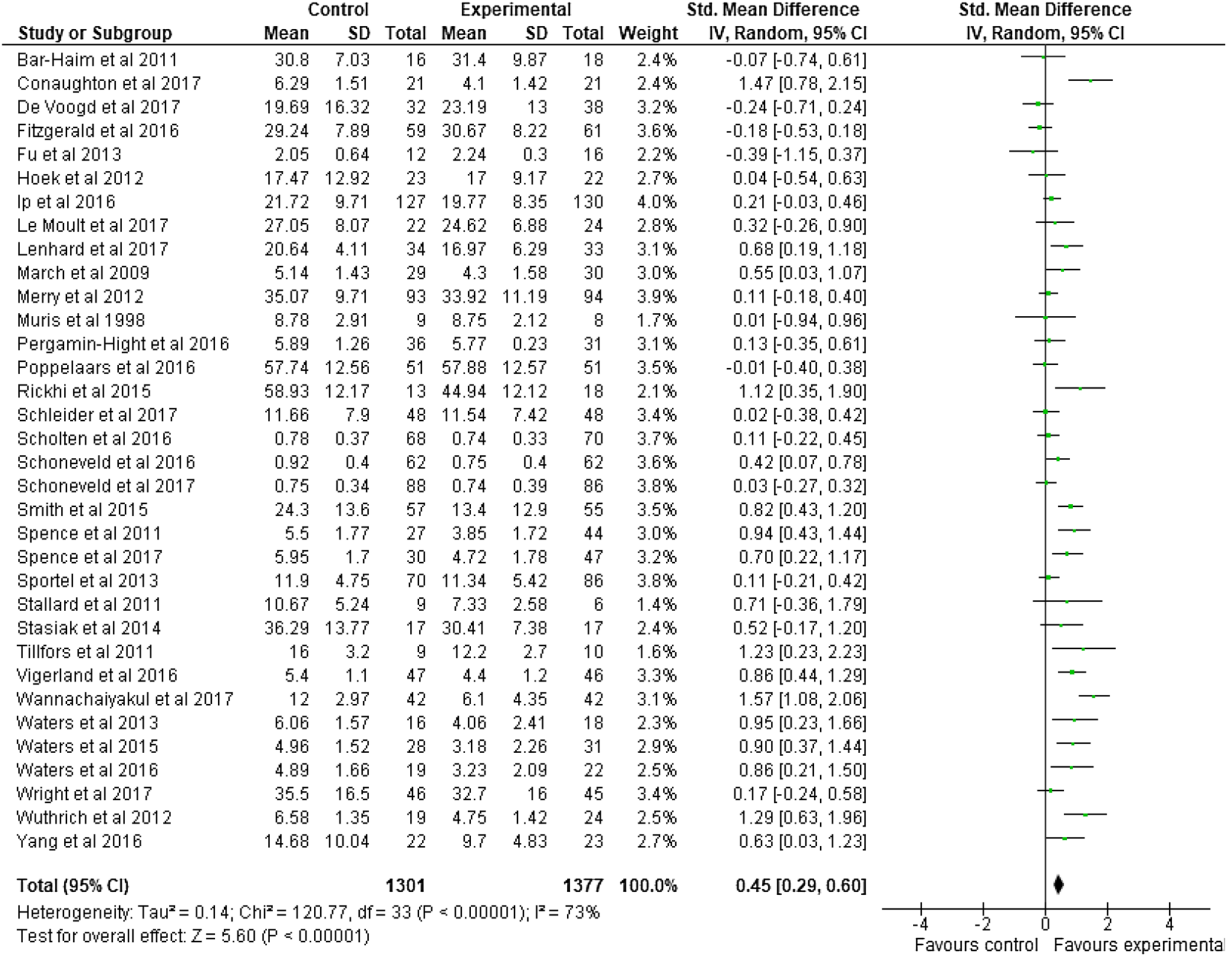
Forest Plot of meta-analysis on technology delivered interventions for depression and anxiety in adolescents compared to control conditions

**Fig. 4:**
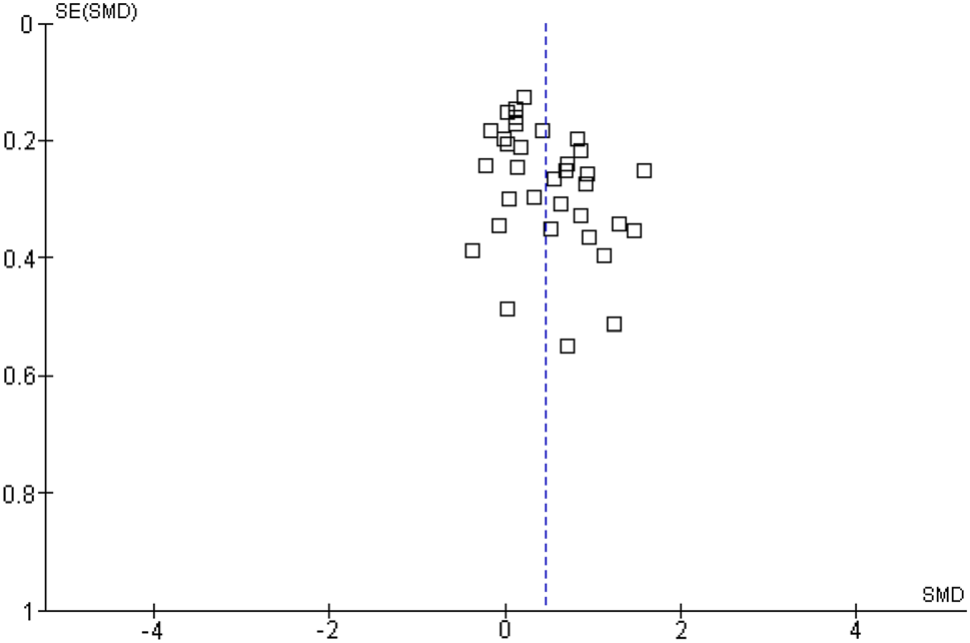
Funnel Plot

A meta-analysis was undertaken to determine the effect of technology delivered interventions on depression and anxiety outcomes compared to wait-list control groups at post-intervention. A random-effects model produced a small overall effect in favor of technology delivered interventions: *g* = 0.45 [95% CI 0.29, 0.60]. The associated Z score was significant (*Z* = 5.60, *p* < 0.00001). Heterogeneity was high and statistically significant (*I*^2^ = 73%, *Q* = 120.77, *df* = 33, *p* < 0.00001). Figure 3 is a forest plot of these results. The associated funnel plot (Fig. 4) is slightly asymmetrical, indicating possible publication bias.

### Sub-group Analyses

#### Age

A sub-group analysis was conducted to determine whether effect sizes varied according to participant age. This analysis showed no significant difference (*Q* = 0.36, *df* = 2, *p* = 0.84) between studies exclusively focused on children (*n* = 3, *g* = 0.58, 95% CI − 0.01 to 1.18, *p* = 0.05), adolescent only samples (*n* = 18, *g* = 0.42, 95% CI 0.21–0.64, *p* < 0.001), or mixed samples (*n* = 13, *g* = 0.39, 95% CI 0.15–0.63, *p* < 0.001).

### Control Condition

A sub-group analysis was performed to examine whether effect sizes varied according to the type of control group the intervention was compared to. There was a statistically significant difference (*Q* = 20.70, *df* = 3, *p* < 0.001) in effect sizes according to category of control group. Technology-based interventions did not produce statistically significant benefits over face to face CBT interventions (*n* = 4, *g* = 0.11 [− 0.06 to 0.28] *p* = 0.92) or other therapy control conditions (*n* = 3, *g* = 0.07 [− 0.15 to 0.30], *p* = 0.52). Technology-based interventions produced a small effect size demonstrating benefit over attention and placebo controls (*n* = 14, g = 0.29 [0.05–0.53], *p* = 0.02) and a medium effect size demonstrating benefit compared to wait-list controls (*n* = 17, *g* = 0.68 [0.47–0.90], *p* ≤ 0.001).

### Mental Health Problem

The following sub-group analysis investigated whether effect sizes differed according to the type of mental health problem, depression or anxiety. There was no significant difference (*Q* = 0.04, *df* = 1, *p* = 0.83) in effect sizes between interventions targeting depression (*n* = 13, *g* = 0.43 [95% CI 0.18–0.68]) and interventions targeting anxiety (*n* = 213, *g* = 0.41 [95% CI 0.12–0.71]).

### Problem Severity

We explored whether diagnostic status influenced effect sizes. There was a significant difference (*Q* = 13.44, *df* = 1, *p* < 0.001) between interventions involving participants with a primary diagnosis of depression or an anxiety disorder (*n* = 18, *g* = 0.72 [95% CI 0.52–0.91] *p* < 0.001) and those involving participants with elevated symptoms of depression or anxiety (*n* = 16, *g* = 0.22 [95% CI 0.03–0.40] *p* = 0.02). Interventions that involved participants with diagnosed disorders had larger effect sizes.

### Theoretical Basis

Studies were grouped according to the theoretical basis of the intervention, including CBT, ABMT, CBM and ‘other’. Sub-group analysis demonstrated a statistically significant difference (*Q* = 11.61, *df* = 3, *p* = 0.009) in effect sizes between interventions based on CBT (*n* = 17, *g* = 0.66 [95% CI 0.42–0.90] *p* < 0.001), interventions based on ABMT (*n* = 8, *g* = 0.41 [95% CI 0.08–0.73] *p* = 0.01), CBM interventions (*n* = 3, *g* = 0.09 [95% CI -0.19–0.37] *p* = 0.53), and ‘other’ interventions (*n* = 6, *g* = 0.20 [95% CI -0.03–0.44] *p* = 0.09). Other interventions and CBM interventions did not demonstrate statistically significant benefits over control conditions.

### Therapist Support

We explored whether therapist support influenced effect sizes. There was a significant effect of therapist support on trial effect sizes (*Q* = 27.28, *df* = 2, *p* < 0.001). Minimal contact therapy produced larger effect sizes (*n* = 9, *g* = 0.87 [95% CI 0.68, 1.06] *p* < 0.001), than predominantly self-help (*n* = 2, *g* = 0.81 [− 0.68, 2.31] *p* = 0.29) and purely self-administered interventions (*n* = 23, *g* = 0.24 [0.10, 0.38], *p* < 0.001).

### Parental Involvement

The next sub-group analysis investigated whether parental support of the intervention influenced effect sizes. Results showed a statistically significant difference (*Q* = 24.43, *df* = 1, *p* < 0.001) with parent supported interventions producing larger effect sizes (*n* = 9, *g* = 0.86 [95% CI 0.69, 1.04] *p* < 0.001) than interventions delivered without parent support (*n* = 23, *g* = 0.25 [95% CI 0.09, 0.42] *p* = 0.002).

### Continuation of Other Treatment

Finally, we explored whether continuation of other treatment for depression or anxiety influenced effect sizes. Results demonstrated a significant difference in effect sizes (*Q* = 9.37, *df* = 1, *p* = 0.002) between trials in which continuation of psychological or pharmacological treatment was permitted (*n* = 6, *g* = 0.90 [95% CI 0.68, 1.11], *p* < 0.001) and trials in which no ongoing treatment was provided (*n* = 16, *g* = 0.42 [95% CI 0.20, 0.63]). The provision of ongoing treatment generated larger effect sizes than trials in which ongoing treatment was not permitted. Some studies were excluded from this analysis due to this information not being reported (*n* = 12).

## Discussion

The aim of this study was to provide an up-to-date investigation of the effect of technology delivered interventions for the treatment of depression and anxiety in children and adolescents. Our systematic search identified 34 randomized controlled trials involving 3113 children and adolescents aged 6–18 years of age. Our search failed to identify any studies of emerging technologies such as virtual reality and m-Health applications (apps) developed specifically for children and adolescents with depression and anxiety. Whilst our search results indicate that research in this area is growing, research does not appear to be keeping pace with advances in technological development. Similarly, the 34 studies we identified related to 29 different programs. Nearly all were, therefore, subject to a single evaluation undertaken by the program developer. Where additional evaluations were undertaken (four programs) these had typically been undertaken with the involvement of the program developers highlighting the need for further independent evaluation.

Technological interventions based on CBT programs yielded a medium post-intervention effect compared to waiting list control groups. This highlights the benefit of technology-based interventions for depression and anxiety in children and adolescents. Given the limited capacity and long waiting times for appointments in many specialist child and adolescent mental health services, technological interventions could offer an effective way of increasing timely access to evidence-based interventions. The majority of these interventions required minimal therapist support and could readily be provided for those on long waiting lists.

### Comparison with Previous Work

CBT based technology delivered interventions yielded an effect size (*g* = 0.66) comparable to other meta-analyses which have shown moderate effect sizes for face to face CBT in children (*d* = 0.66; Arnberg and Öst 2014) and for CCBT/ICBT for adolescents (*g* = 0.72; Ebert et al. 2015). Although ABMT based interventions produced a small effect size (*g* = 0.41) this is larger than those reported in previous reviews (*g* = − 0.19, Pennant et al. 2015). As a general update on the research literature since the work of Pennant et al. (2015), our review has found 21 new RCT’s published since 2013. In accordance with Pennant et al’s work, we mostly found CCBT/ICBT and ABMT based programs and failed to find any new RCT’s of Smartphone apps or virtual reality-based programs. Pennant et al.could not be confident in the effect of CCBT on children (defined as 5–11 years). In the present analysis, we demonstrated no significant difference in effect sizes between studies with children (5–11 years), adolescents (12–18 years) and mixed ages (5–18 years) and all sub-groups demonstrated a significant benefit over control groups. However, we only identified three studies involving a total of 67 children which had an exclusive child sample (Bar-Haim et al. 2011; Waters et al. 2015, 2016). Of these, two studies also permitted ongoing treatment and compared ABMT to a wait-list control, factors which in our review significantly moderated outcomes. The evidence to support the use of technological interventions with children under the age of 12 remains limited and as such we cannot be confident in the effects of these interventions with children. Further research is required to investigate the effects of technological interventions on children and the level of parental support that may be beneficial.

### Sub-group Analyses Findings

Technology delivered interventions failed to demonstrate a significant benefit over face to face CBT or other therapy conditions. This finding is in accordance with a previous meta-analysis of ICBT in children and adolescents. This also found no significant difference in effects between ICBT and face to face CBT (Vigerland et al. 2016b). Like Vigerland et al. (2016b) our analysis only includes a small number of studies utilizing face to face CBT as a control (four) which may limit our conclusions. It is also important to note that two out of the four interventions were not based on CBT. Mindlight (Schoneveld et al. 2017) is a videogame incorporating neurofeedback (EEG) training, exposure training and ABM; the other intervention is based on cognitive bias modification (Sportel et al. 2013). Both these approaches have a limited evidence base, particularly in comparison to face to face CBT. In contrast, face to face CBT is not only supported by a large evidence base (Hofmann et al. 2012) but is also considered to be the ‘gold standard’ in psychological treatment (David et al. 2018).

Comparing a technology-based intervention to an attention placebo control produced statistically significant benefits and a small effect size. Comparing technology delivered interventions to wait-list control groups yielded a moderate effect in favor of the intervention. This is consistent with previous analyses which have demonstrated moderate effects using this comparison in adolescent populations (Richards and Richardson 2012; Vigerland et al. 2016b), in adult populations (Grist and Cavanagh 2013) and in comparing face to face CBT with wait-list groups (Hofmann et al. 2012). As such our conclusions are similar to those of Pennant et al. (2015); that evidence for the benefit of technology-based interventions compared to face to face therapies is sparse and should not replace face to face therapy. It does however suggest that technological interventions could offer a low-cost alternative treatment when face to face treatments are not available or feasible.

There was no significant difference in effect sizes between interventions targeting depression and those targeting anxiety, suggesting programs for depression and anxiety were equally effective. In a recent meta-review of digital health interventions for children and young people, Hollis et al. (2017) summarized findings from six meta-analyses (Davies et al. 2014; Ebert et al. 2015; Pennant et al. 2015; Podina et al. 2016; Ye et al. 2014) which demonstrated CCBT for depression yielded small to moderate effect sizes, whereas CCBT for anxiety yielded moderate to large effects in favor of the intervention. Hollis et al. (2017) did not, however, analyze whether these differences were significantly different. The results from the present analysis would suggest they may not be statistically, or meaningfully different.

Sub-group analyses also indicated that technological interventions involving participants with a confirmed diagnosis of depression or anxiety produced significantly larger effect sizes than interventions trialed with populations experiencing elevated symptoms. Few previous meta-analyses have explored the effect of problem severity on effect sizes and those that have, yield contrasting results. Ebert et al’s (2015) analysis found no significant difference in effect sizes between trials involving participants (up to age 25) with a ‘confirmed diagnosis’ (*n* = 6; *g* = 0.71) and those with ‘anxiety/depressive symptoms’ (*n* = 7, *g* = 0.74) although this focused only on computer and internet CBT. The analysis by Pennant et al. (2015) found a significant effect for anxiety but not for depression although the authors note that sample sizes were small. Our findings contrast with these reviews but are consistent with findings involving adults. Analyses of outcome predictors for iCBT for depression in adults have demonstrated that higher pre-treatment symptom severity is related to greater symptom reduction (Button et al. 2012; Edmonds et al. 2018). This finding tends to be explained in terms of individuals with greater symptom severity having greater room for improvement and symptom reduction (Edmonds et al. 2018). This may explain why effect sizes in our analysis were larger for diagnosed groups. Future research should explore the role of problem severity in treatment outcomes to determine for whom technology delivered interventions will be beneficial.

Our review found that interventions based on CBT produced the largest effect sizes, followed by interventions based on ABMT. The potential benefit of CBT based interventions adds to the conclusions of previous reviews of ICBT/CCBT (Ebert et al. 2015; Pennant et al. 2015; Podina et al. 2016; Vigerland et al. 2016a) and is expected given the developed evidence-based for CBT as an intervention for depression and anxiety (Cuijpers et al. 2013; Watts et al. 2015). As noted previously, CBT is considered the ‘gold standard’ psychological therapy (David et al. 2018). Interestingly, our finding that ABMT produced small, significant effects diverges from the findings of Pennant et al. (2015) for whom three ABMT studies produced no significant benefit over control conditions. However, our conclusions remain similar; while there is some indication that ABMT may be effective, we cannot be confident in the demonstrated benefit of ABMT as studies and the overall effect sizes were small. Both CBM based interventions and interventions classified as ‘other’ (problem-solving therapy, growth mindset, bio/neurofeedback emotion regulation videogames) failed to demonstrate a significant benefit over control conditions. This may reflect the limited research which has been undertaken but at this stage, we cannot be confident in the benefit of these interventions.

Previous evidence demonstrates mixed results regarding the role of therapeutic support in e-Health interventions. In adult populations, meta-analyses consistently demonstrate that therapist supported programs produce larger effect sizes than programs that provide no therapist support (Johansson and Andersson 2012; Richards and Richardson 2012). For children and adolescents, findings from Pennant et al. (2015) indicated no significant difference between programs for depression with ‘minimal’ vs. ‘some’ therapist input. Other evidence suggests that for programs targeting anxiety, ‘minimally’ supported interventions yield larger effect sizes than ‘significantly’ supported programs (Podina et al. 2016). Our analysis found that effect size was related to therapeutic support, specifically, minimal contact therapies (more than 90 min) yielded higher effect sizes than purely self-administered interventions. In traditional face to face therapies, a body of research has supported the role of ‘common factors’, such as the working alliance, in producing positive therapeutic outcomes (Lambert 1992). It is logical therefore that the more opportunity for interaction there is with a therapist in technology-based interventions, the more scope there is to build a therapeutic relationship, thereby improving outcomes. Therapeutic support has also been identified as a moderating factor influencing engagement as well as therapeutic outcomes (Hollis et al. 2017; Rickwood and Bradford 2012). Therefore, therapeutic support is also likely to improve engagement and motivation to continue using technology-based interventions. These findings extend those of Richards and Richardson (2012) to a child and adolescent population.

The importance of parental support in CCBT and technology delivered interventions is poorly understood (Vigerland et al. 2016b). The parental input may range from assisting children and adolescents in setting up the program to active participation in program sessions. Our sub-group analysis showed parent supported interventions produced significantly larger effect sizes than interventions delivered without, although both types of programs were significantly better than control groups. These results contrast with those of Ebert et al. (2015). These divergent results may be explained by the differences in study eligibility criteria previously described. Our results do however corroborate previous work with face to face CBT in children which suggests treatment outcomes can be improved with parental involvement (Barrett et al. 1996; Creswell and Cartwright-Hatton 2007). It has been suggested that parental involvement may aid the successful learning and application of new skills, perspectives and applying specific strategies learned in therapy into real life (Siddaway et al. 2014; Spence et al. 2000; Stallard 2005; Thirlwall et al. 2013). As such their children might be more likely to continue to understand and implement therapeutic strategies and so make therapeutic gains (Thirlwall et al. 2013). Parental involvement may also aid changes in family processes and parental risk factors implicated in the development and maintenance of childhood anxiety (Barrett et al. 1996; Siddaway et al. 2014).

As far as we are aware, no previous meta-analysis of e-Health in children and adolescents with depression or anxiety has explored whether continuation of other psychological or pharmacological treatment for depression or anxiety during the trial influences effect sizes. Our sub-group analysis demonstrated significant differences between trials permitting ongoing treatment and those explicitly forbidding ongoing psychological and/or pharmacological treatment. Trials permitting ongoing treatment produced a large effect size, whereas trials forbidding ongoing treatment yielded a small effect size, both significant. It is possible that for the ongoing treatment permitted studies, the larger effect sizes are due to a ‘combination effect’ of the program, plus any ongoing treatment, or they could be due to either the program or ongoing intervention alone. While some previous work has shown that ‘combined treatment’ is superior to psychological interventions or pharmacological interventions in adults (Cuijpers et al. 2014), for children this is uncertain (Cox et al. 2014; Walkup et al. 2008). It is important to acknowledge that only three out of the six studies (Lenhard et al. 2017; Rickhi et al. 2015; Smith et al. 2015) actually reported the number of participants who received an ongoing psychological or pharmacological treatment during the study intervention. The remaining three only noted in the inclusion criteria that ongoing treatment was permitted (Tilfors et al. 2011; Vigerland et al. 2016a; Wuthrich et al. 2012). Therefore, we cannot be certain of the extent or nature of the additional ongoing treatment that was provided. Our conclusions about the benefits of using technology alongside ongoing interventions requires further exploration and should be interpreted cautiously.

Finally, as with previous reviews (Ebert et al. 2015; Pennant et al. 2015), most RCT’s were conducted in high-income countries and we cannot, therefore, generalize these results to low- or middle-income countries. The need for access to evidence-based mental health interventions in low-income countries is considerable and the potential of technology delivered interventions in meeting this need will not be fulfilled until future research is conducted in these contexts.

### Limitations

While our results demonstrate the potential benefit of technology delivered interventions, our review does have a number of limitations: First, our analyses are limited to post-intervention outcomes only and we were unable to assess whether these immediate benefits persist into the medium term. Second, our main analysis compared technological interventions with wait-list control groups assessed after a specified period of time. Whilst this provides a consistent timeframe for comparisons some participants may not have completed the intervention by follow-up. Reporting of program completion rates was poor and were unavailable for 12 studies. Where we were able to extract them the average program completion rate of 64% indicates that a number of participants had not completed the program. Third, the included studies suffered from several methodological limitations. Study protocols were seldom reported, and sample sizes were small with 14 reporting a sample of fewer than 50 participants. Although there was significant bias regarding the non-blinding of participants this is inevitable when comparing an intervention to a waiting list condition. Ethical practice requires participants to be fully aware of the study conditions and as such it is impossible to effectively blind participants to treatment allocation (Button et al. 2015). Finally, our search strategy was limited to published papers. Given the speed of growth in this area, it is probable that further studies might have been identified.

## Future Directions

While the presented studies offer an insight into the potential benefit of technology delivered programs, future work requires better quality trial design and reporting; the use of adequate sample sizes, appropriate active control groups as well as adequate reporting of program completion and attrition. Well-designed RCT’s will also be required to determine the sole effect of ICBT/CCBT distinct from combined effects with ongoing psychological or pharmacological treatment. Adequate investigation and reporting of the cost-effectiveness of technology-based interventions are also necessary, particularly because cost-effectiveness is one of the main proposed benefits of technology-based interventions (Vigerland et al. 2016b).

As far as can be determined from the information reported, none of the programs trialed included adolescents in program design. Recent work investigating a m-Health intervention for children and adolescents who self-harm (Stallard et al. 2018) demonstrated the value of involvement of adolescents with lived experience in the design of interventions, particularly for program acceptability. Given the high level of attrition from e-Health interventions (Melville et al. 2010) it would be beneficial for future work to explore the impact of program co-production on engagement and attrition.

## Conclusions

This meta-analysis provides a unique update on the current evidence for the effect of technology-based interventions in children and adolescents. Our systematic search identified 34 RCTs examining technology delivered interventions (primarily based on CBT and ABMT) for depression or anxiety in youth populations. Overall, the analysis provides support for the effectiveness of CBT based technology delivered interventions for both depression and anxiety in comparison to wait-list controls. Interventions based on ABMT yielded only a small effect size and CBM and ‘other’ programs (problem-solving therapy, growth mindset, bio/neurofeedback emotion regulation videogames) failed to demonstrate a significant benefit over control groups. We, therefore, cannot be confident in the benefit of these interventions at this stage.

Therapist supported, and parent supported programs produced better outcomes. Given that therapist-assisted programs produced better outcomes and comparisons to face to face CBT did not confer any significant benefit, our overall conclusions are consistent with previous work (Hollis et al. 2017; Pennant et al. 2015): The current evidence base does not support the use of technology delivered interventions as a replacement for face to face psychological interventions. However, the magnitude of effects demonstrated suggest there is a benefit in using technology delivered CBT interventions where access to face to face CBT and other psychotherapies are limited or delayed.

## Appendix A: Search Strings by Databases

APA PsychNet

27/09/17

254 Results for Any Field: CCBT OR Any Field: “computer assisted therapy” OR Any Field: computerised OR Any Field: computerized OR Any Field: computer OR Any Field: “CD-ROM” OR Any Field: “DVD-ROM” OR Any Field: “mHealth*” OR Any Field: “mobile health” OR Any Field: “mobile device” OR Any Field: “mobile app” OR Any Field: “smartphone” OR Any Field: “mobile phone” OR Any Field: Internet OR Any Field: “ICBT” OR Any Field: web OR Any Field: “virtual reality” AND Any Field: “depress*” OR Any Field: “self – harm” OR Any Field: “suicid*” OR Any Field: anxi* OR Any Field: “PTSD” OR Any Field: “social anxi*” OR Any Field: “separation anxi*” OR Any Field: phobia OR Any Field: “generalised anxiety disorder” OR Any Field: “OCD” AND Any Field: child* OR Any Field: teenage* OR Any Field: adolescen* OR Any Field: “young per*” OR Any Field: youth* AND Any Field: “randomized controlled trial” OR Any Field: randomized OR Any Field: randomised AND Age Group: Preschool Age (2-5 yrs) OR School Age (6-12 yrs) OR Adolescence (13-17 yrs) AND Document Type: Journal Article AND Year: 2013 To 2018

Embase

27/09/17

189 results for search (ccbt OR ‘computer assisted therapy’/exp OR ‘computer assisted therapy’ OR computerised OR computerized OR ‘computer’/exp OR computer OR ‘cd-rom’/exp OR ‘cd-rom’ OR ‘dvd-rom’ OR mhealth* OR ‘m-health*’ OR ‘mobile health’/exp OR ‘mobile health’ OR ‘mobile device’/exp OR ‘mobile device’ OR ‘mobile app’/exp OR ‘mobile app’ OR ‘smartphone’/exp OR smartphone OR ‘mobile phone’/exp OR ‘mobile phone’ OR ‘internet’/exp OR internet OR icbt OR ‘web’/exp OR web OR vr OR ‘virtual reality’/exp OR ‘virtual reality’) AND (depress* OR ‘self-harm’ OR suicid* OR anxi* OR ‘ptsd’/exp OR ptsd OR ‘social anxi*’ OR ‘separation anxi*’ OR ‘phobia’/exp OR phobia OR ‘generalised anxiety disorder’/exp OR ‘generalised anxiety disorder’ OR ocd) AND (child* OR teenage* OR adolescen* OR ‘young per*’ OR youth* OR ‘young adult*’) AND (‘randomized controlled trial’/exp OR ‘randomized controlled trial’ OR randomised) AND [2013-2017]/py AND ‘randomized controlled trial’/de AND ‘article’/it AND ([adolescent]/lim OR [child]/lim OR [preschool]/lim OR [school]/lim)

Google Scholar

27/09/17

31 results for Computerized CCBT mHealth “smartphone app” “virtual reality” “mental health” child adolescent “randomized controlled trial”

PubMed

27/09/17

254 results for

((((ccbt OR “computer assisted therapy” OR computerized OR computerized OR computer OR cd-rom OR dvd-rom OR “mHealth*” OR “m-health*” OR “mobile health” OR “mobile device” OR “mobile app” OR “smartphone” OR “mobile phone” OR internet OR icbt OR web OR “virtual reality”)) AND (“depress*” OR “self - harm” OR suicid* OR anxi* OR “PTSD” OR “social anxi*” OR “separation anxi*” OR phobia OR “generalised anxiety disorder” OR “OCD”)) AND (child* OR teenage* OR adolescen* OR “young per*” OR youth*)) AND (“randomized controlled trial” OR randomized OR randomised) Filters activated: Publication date from 2013/01/01 to 2018/12/31, Child: birth-18 years

**Science Direct**

Search results: 67 results found for pub-date > 2012 and TITLE-ABSTR-KEY(Computerized OR CCBT OR mHealth OR “smartphone app” OR “virtual reality”) and (“mental health” AND child OR adolescent AND “randomized controlled trial”).

**Scopus**

27/09/17

909 results for

(ccbt OR “computer assisted therapy” OR computerised OR computerized OR computer OR cd-rom OR dvd-rom OR “mHealth*” OR “m-health*” OR “mobile health” OR “mobile device” OR “mobile app” OR “smartphone” OR “mobile phone” OR internet OR icbt OR web OR “virtual reality”) AND (“depress*” OR “self -harm” OR suicid* OR anxi* OR “PTSD” OR “social anxi*” OR “separation anxi*” OR phobia OR “generalised anxiety disorder” OR “OCD”) AND (child* OR teenage* OR adolescen* OR “young per*” OR youth* OR “young adult*”) AND (“randomized controlled trial”) AND (LIMIT-TO (SRCTYPE, “j ")) AND (LIMIT-TO (DOCTYPE, “ar ")) AND (LIMIT-TO (SUBJAREA, “PSYC ")) AND (LIMIT-TO (PUBYEAR, 2018) OR LIMIT-TO (PUBYEAR, 2017) OR LIMIT-TO (PUBYEAR, 2016) OR LIMIT-TO (PUBYEAR, 2015) OR LIMIT-TO (PUBYEAR, 2014) OR LIMIT-TO (PUBYEAR, 2013)) AND (LIMIT-TO (LANGUAGE, “English ")) AND (LIMIT-TO (EXACTKEYWORD, “Controlled Study ") OR LIMIT-TO (EXACTKEYWORD, “Adolescent ")) AND (LIMIT-TO (EXACTKEYWORD, “Adolescent”))

**Social Policy and Practice**

**27 results**

27/09/2016

((CCBT or “computer assisted therapy” or computerised or computerized or computer or CD-ROM or DVD-ROM or mHealth or “m-health” or “mobile health” or “mobile device” or “mobile app” or smartphone or “mobile phone” or Internet or ICBT or web or “virtual reality”) and (depression or “self-harm” or suicide or anxiety or “PTSD” or “social anxiety” or “separation anxiety OR phobia” or “generalised anxiety disorder” or “OCD” or “conduct disorder” or “eating disorder” or anorexia or bulimia or “binge eating” or “body image” or “mental health” or schizophrenia or “bipolar affective disorder” or psychosis or insomnia or ADHD or Autism or substance) and (child* or teenage* or adolescent or “young person” or youth* or “young adult”) and (“randomized controlled trial” or randomized or randomised)).af. limit 1 to yr=“2013-Current”

**Web of Science**

**27**/**09**/**2017**

**Results: 361**

*(from Web of Science Core Collection)*

**You searched for: TOPIC**: (CCBT OR computer assisted therapy OR Computerised OR computerized OR computer OR CD-ROM OR DVD-ROM OR mHealth* OR m-health* OR mobile health OR mobile device OR mobile app OR smartphone OR mobile phone OR Internet OR ICBT OR web OR virtual reality) *AND***TOPIC**: (depress* OR self harm OR suicid* OR anxi* OR PTSD OR social anxi* OR separation anxi*OR phobia OR generalised anxiety disorder OR OCD) *AND***TOPIC**: (child* OR teenage* OR adolescen* OR young per* OR youth*) *AND***TOPIC**:(“randomized controlled trial”)

**Timespan**: 2013-2017. **Indexes**: SCI-EXPANDED, SSCI, A&HCI, ESCI.

**JMIR**

27/09/17

33 results

Abstract Computerized CCBT mHealth “smartphone app” “virtual reality” All fields depression OR anxiety AND child adolescent AND “randomized controlled trial”

Cyberpsychology, Behavior and Social Networking

26/09/17

6 RESULTS

You searched for: [[All: “computerized”] OR [All: ccbt] OR [All: mhealth] OR [All: “smartphone]] AND [[All: app”]OR [All: “virtual]] AND [All: reality”] AND [All: “mental health”] AND [[All: child] OR [All: adolescent]] AND [Publication Date: (01/01/2013 TO 12/31/2017)] AND [[Categories: Psychology, Humanities, and Social Science]OR [Book/Issue: Advances in Preschool Psychopharmacology] OR [in Journal: Journal of Child and Adolescent Psychopharmacology] OR [in Journal: Violence and Gender] OR [in Journal: Cyberpsychology, Behavior, and Social Networking]]

6 articles matched your search criteria.
